# *De novo* European eel transcriptome provides insights into the evolutionary history of duplicated genes in teleost lineages

**DOI:** 10.1371/journal.pone.0218085

**Published:** 2019-06-12

**Authors:** Christoffer Rozenfeld, Jose Blanca, Victor Gallego, Víctor García-Carpintero, Juan Germán Herranz-Jusdado, Luz Pérez, Juan F. Asturiano, Joaquín Cañizares, David S. Peñaranda

**Affiliations:** 1 Grupo de Acuicultura y Biodiversidad, Instituto de Ciencia y Tecnología Animal, Universitat Politècnica de València, Camino de Vera s/n, Valencia, Spain; 2 Instituto de Conservación y Mejora de la Agrodiversidad Valenciana, Universitat Politècnica de València, Valencia, Spain; Universite de Rouen, FRANCE

## Abstract

Paralogues pairs are more frequently observed in eels (Anguilla sp.) than in other teleosts. The paralogues often show low phylogenetic distances; however, they have been assigned to the third round of whole genome duplication (WGD), shared by all teleosts (3R), due to their conserved synteny. The apparent contradiction of low phylogenetic difference and 3R conserved synteny led us to study the duplicated gene complement of the freshwater eels. With this aim, we assembled *de novo* transcriptomes of two highly relevant freshwater eel species: The European (*Anguilla anguilla*) and the Japanese eel (*Anguilla japonica*). The duplicated gene complement was analysed in these transcriptomes, and in the genomes and transcriptomes of other Actinopterygii species. The study included an assessment of neutral genetic divergence (4dTv), synteny, and the phylogenetic origins and relationships of the duplicated gene complements. The analyses indicated a high accumulation of duplications (1217 paralogue pairs) among freshwater eel genes, which may have originated in a WGD event after the Elopomorpha lineage diverged from the remaining teleosts, and thus not at the 3R. However, very similar results were observed in the basal Osteoglossomorpha and Clupeocephala branches, indicating that the specific genomic regions of these paralogues may still have been under tetrasomic inheritance at the split of the teleost lineages. Therefore, two potential hypotheses may explain the results: i) The freshwater eel lineage experienced an additional WGD to 3R, and ii) Some duplicated genomic regions experienced lineage specific rediploidization after 3R in the ancestor to freshwater eels. The supporting/opposing evidence for both hypotheses is discussed.

## Introduction

Large accumulations of gene duplications can originate from one single event, like a whole genome duplication (WGD) [1] or from multiple small duplication events such as small segmental duplications (SDs) [2], which are often found in tandem. Any of these duplication events may contribute to species evolution by providing raw genetic material for new phenotypic variation [1–3].

Relatively recent SDs are often found in tandem and have been found in high abundance in several organisms including yeast [4], daphnia [5], humans [2,6,7] and teleosts [8–12]. Soon after a SD, one paralogue is most commonly lost [1] possibly due to an accumulation of deleterious mutations or genetic drift [13]. In a few cases, a high abundance of SDs can persist for millions of years as seen in yeast [4], common carp [9] and humans [2,6,14]. This process has been associated with adaptation to new environments [5,15,16]. On the other hand, WGDs are presumed rare in mammals [17], but are recurrently found in amphibians and reptiles [18] and have frequently been suggested in insects [18], fungi [19], and plants [20–23]. Recent WGD events have traditionally been observed by cytological studies through the observation of additional chromosomes [22]; however, ancient WGD events are often hidden [22,24–26] by massive gene losses [27–29] and the fusion or loss of duplicated chromosomes [19,30–33]. Therefore, an ancient WGD event can only be discovered through specific analysis at a whole genome level. Consequently, discoveries of WGD events have accelerated as sequencing techniques have improved and genome-scale data has become more accessible [22,24,26]. It has been suggested that early on in the vertebrate lineage two WGDs (1R and 2R) occurred resulting in species radiation and evolution of new traits [1–3,34]. In teleosts, strong genomic evidence supports the existence of an additional WGD called the teleost specific 3^rd^ round of WGD (3R), which occurred in the base of the teleost lineage between 350 and 320 million years ago (MYA) [35,36].

In addition to 3R, WGD events appear to be a reoccurring phenomenon in Actinopterygii even when only considering cytological evidence [37,38]. Furthermore, Inoue *et al*. [27] found that 70–80% of the genes originating from the 3R WGD get lost after just 60 million years. Similarly, other studies have found that in most teleosts 3–20% of the genes generated during 3R are conserved today [31]. Moreover, extensive chromosome reorganizations have been suggested in the teleost lineage associated with 3R [39,40] and after the salmonid specific 4^th^ round of WGD (Ss4R) [41]. Therefore, it has been suggested that further discoveries of new WGDs in teleosts may increase following the development of sequencing techniques and the increase in the number of studies specifically analysing the temporal distribution and quantity of gene duplications [31]. This phenomenon of accelerating rates of WGD discoveries is currently observed in plant genomics [20–22,42].

Following a WGD event, paralogues genes will start to diverge after the recombination between duplicated genes has stopped at the transition from tetrasomic to disomic inheritance [41,43,44], also referred to as cytological rediploidization. However, after autotetraploidization tetrasomic segregation may continue due to the high similarity between the duplicated chromosomes, and thus rediploidization may be vastly delayed after a WGD event [43]. Therefore, variations in phylogenetic divergences between paralogue gene pairs originating from the same WGD event can appear in cases where a genomic region is under tetrasomic inheritance, at the time of a speciation event [43]. The resulting phylogenetic gene family trees from such event are virtually indistinguishable from gene trees where additional gene duplications have occurred [44]. In particular, in salmonids, strong evidence suggests that rediploidization after the Ss4R has been protracted in time for approximately a quarter of the genome [41,45]. In turn, this mechanism has led to several salmonid gene duplicates to not present 1:1 orthology relationships among different salmonid species, despite being created at the Ss4R [41,46,47]. A protracted pseudotetraploid period has also been suggested in teleosts after 3R [44]. In particular, the peculiar hox gene complement of the African butterfly fish (*Pantodon buchholzi*) is most parsimoniously explained by a hypothesis which includes protracted rediploidization for some genomic regions [44]. However, unequivocal support of protracted rediploidization beyond salmonids will require further careful phylogenomic analysis [43].

Several studies have revealed a high occurrence of duplicated genes in freshwater eels (*Anguilla* spp., Elopomorpha) [48–56]. While these duplicated genes often present weak conserved synteny, suggesting a 3R origin, they also present low phylogenetic divergence between paralogues, indicating that they recently started to diverge. For example, Lafont *et al*. [50] hypothesize that the entire genomic region containing the gene *gper* could have been duplicated in freshwater eels, and maybe also in other teleosts; and that the retention of duplicated genes may be higher in these eels than in other teleosts.

The occurrence of duplicated genes in freshwater eels seems to be higher than for most teleost lineages, and specifically, the remarkably high conservation of duplicated gene sequences since 3R, often hypothesized for freshwater eel genes [48–56], would be unique [57]. Owing to the fact that the availability of genetic raw material has been suggested to increase the potential of novel adaptation [42], information on the duplicated gene complement of eels may prove valuable in understanding the biology of these endangered species. Therefore, the peculiarity of the published data led us to quantify and analyse duplications in the most relevant freshwater eel species and investigate the temporal distribution of the events that created them. To this end, we assembled *de novo* transcriptomes of Japanese (*Anguilla japonica)* and European eel (*Anguilla anguilla)* from downloaded and newly generated Illumina RNA sequencing data, respectively. Furthermore, we performed phylogenetic reconstructions, assigned paralogue pairs to branches of the resulting species tree, and calculated fourfold synonymous third-codon transversion (4dTv) distances for each paralogue pair identified within these transcriptomes. These analyses were run on our *de novo* transcriptomes and on multiple other fish transcriptomes and genomes. Our analysis supports the commonly suggested hypothesis of a high abundance of paralogue pairs, unique to the freshwater eel species. However, the phylogenetic and 4dTv analyses suggest a post 3R origin, and a strong signal of synteny between the genomic environments of these paralogues opposes a hypothesis of a SD origin. Similar results were also obtained from the included Osteoglossomorpha branches and the basal Clupeocephala branch. This, in turn, suggests that the results were generated by protracted rediploidization in teleosts after the 3R. These results thus open a discussion on whether these duplicated genes are the result of a 4R WGD in a common ancestor to freshwater eels or rather have been conserved on chromosomal regions, which have experienced delayed rediploidization after the 3R.

## Materials and methods

### Fish husbandry

Ten immature farm European eel males (mean body mass 96.7±3.6 g ± SEM) supplied by Valenciana de Acuicultura S.A. (Puzol, Valencia, Spain) were transported to the Aquaculture Laboratory at the Universitat Politècnica de València, Spain. The fish were kept in a 200-L tank, equipped with individual recirculation systems, a temperature control system (with heaters and coolers), and aeration. The fish were gradually acclimatized to seawater (final salinity 37 ± 0.3‰), over the course of two weeks. The temperature, oxygen level and pH of rearing were 20°C, 7–8 mg/L and ~ 8.2, respectively. The tank was covered to maintain, as far as possible, a constant dark photoperiod, and the fish were starved throughout the holding period. After acclimation, the fish were sacrificed in order to collect samples of the forebrain (telencephalon, diencephalon, and olfactory bulb), pituitary, and testis tissues.

### Human and animal rights

This study was carried out in strict accordance with the recommendations given in the Guide for the Care and Use of Laboratory Animals of the Spanish Royal Decree 53/2013 regarding the protection of animals used for scientific purposes (BOE 2013), and in accordance with the European Union regulations concerning the protection of experimental animals (Dir 86/609/EEC), Guidelines of the European Union (2010/63/EU). The protocol was approved by the Experimental Animal Ethics Committee from the Universitat Politècnica de València (UPV) and final permission was given by the local government (Generalitat Valenciana, Permit Number: 2014/VSC/PEA/00147). The fish were sacrificed using an overdose of anaesthesia.

### RNA extraction and sequencing

High quality RNA was extracted from the forebrain, pituitary, and testis samples of one individual male eel (weight: 105.4 g, length: 38.5 cm, and eye index: 4.62), following the protocol developed by Peña-Llopis and Brugarolas [58]. The quantity and quality were tested using a bioanalyser (Agilent Technologies, USA), the samples with sufficient RNA integrity number (RIN) values (RIN > 8.2) and RNA amounts (>3 μg of total RNA) were selected. Total RNA of the three samples were shipped to the company Macrogen Korea (Seoul, South Korea). mRNA purification was carried out on these samples, using Sera-mag Magnetic Oligo (dT) Beads, followed by buffer fragmentation. Reverse transcription was followed by PCR amplification to prepare the samples for sequencing using the TruSeq stranded mRNA LT sample prep kit (Illumina, San Diego, USA). The strand information was kept in an Illumina Hiseq-4000 sequencer (Illumina, San Diego, USA). Resulting raw sequences were 101bp paired-end reads which are available at the NCBI Sequence Read Archive (SRA) under the accession no. SRP126643.

### Transcriptome assemblies and genomes

**Fig 1:**
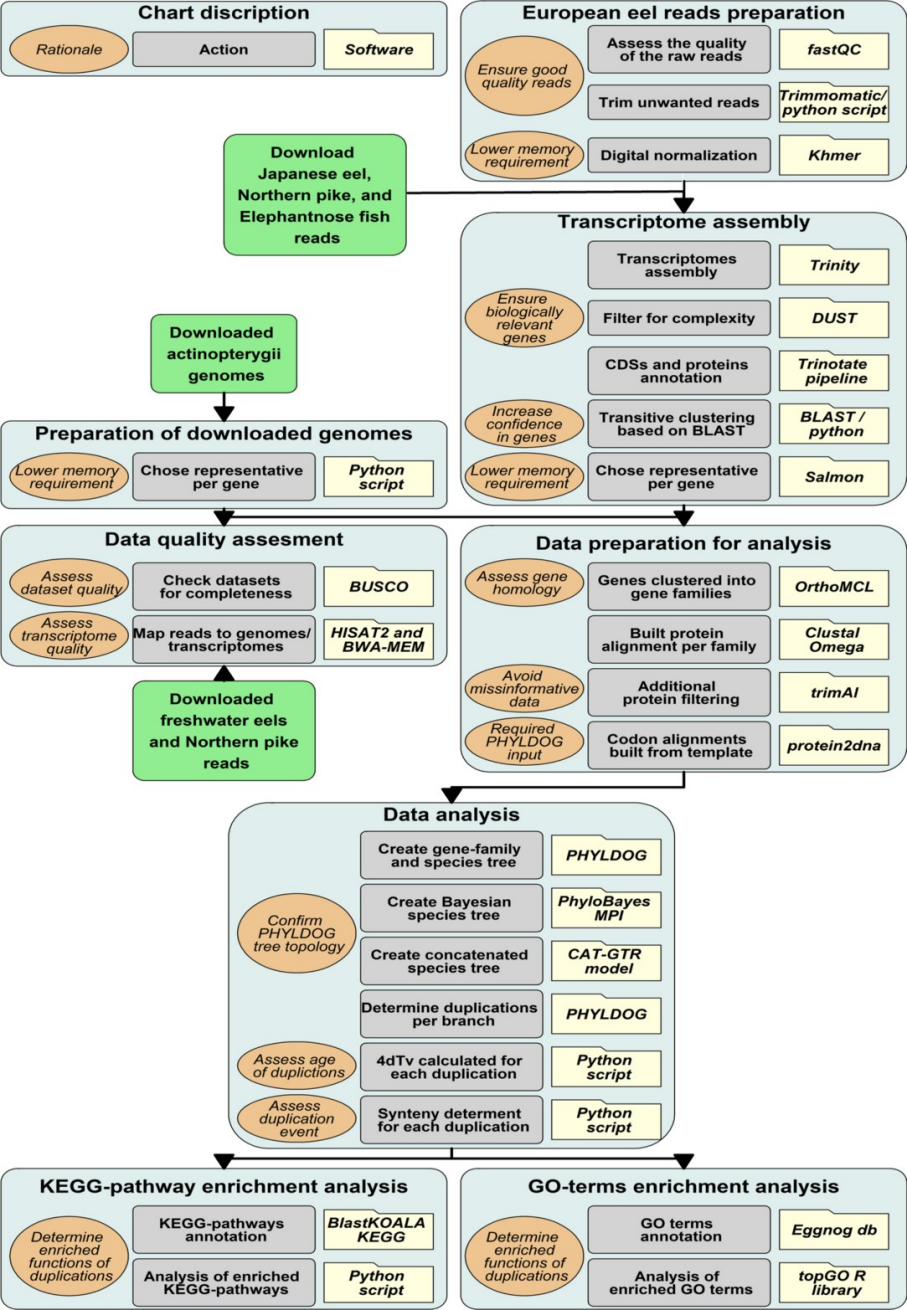
Methodology. Pipeline of the bioinformatics methodology. Folders describe the software used, light grey boxes describe the action taken, light brown bubbles describes the rationale for selected actions, and light blue boxes describe the specific goal of each section. Finally, green boxes represent external data input.

The bioinformatics methodology described below is illustrated in Fig 1. Specifically, FastQC [59] software was used to assess the quality of the raw reads generated by Macrogen. Thereafter, trimmomatic [60] was used to trim the reads, eliminating known adaptor sequences, and low quality regions. Finally, trimmed reads shorter than 50 bp were filtered out. European eel reads were digitally normalized before assembly by Khmer software [61] using a k-mer length of 25 and a coverage of 100. Furthermore, the RNA-Seq raw reads of a Japanese eel Fertilized egg (SRA, NCBI: SRR1930110), preleptocephalus (SRA, NCBI: SRR1930112), leptocephalus (SRA, NCBI: SRR1930115) and glass eel (SRA, NCBI: SRR1930117) were downloaded from NCBI. The RNA-Seq raw reads for Northern pike (*Esox lucius*), elephantnose fish (*Gnathonemus petersii*) and silver arowana (*Osteoglossum bicirrhosum*) were downloaded from the PhyloFish project [62]. All transcriptomes were then assembled using Trinity software [63], taking the strand orientation (for European eel) into account. Naturally produced transcripts may include intervals with a high bias for specific nucleotides (low-complexity), such transcripts may give high-scoring blast results but in fact be biologically insignificant. Therefore, the transcripts assembled were filtered according to their complexity (with a DUST score threshold of 7 and a DUST window of 64), length (with a minimum length of 500 bp), and level of expression (with a transcripts per million (TPM) threshold of 1). The DUST module from BLAST (http://blast.ncbi.nlm.nih.gov/Blast.cgi) was used for this filtering, and Salmon software [64] was used to estimate TPM. After assembly, the coding DNA sequences (CDSs) and proteins were annotated using the Trinotate functional annotation pipeline [63]. Transcripts that share k-mers were clustered by Trinity. However, these transcripts might correspond to different transcript forms of the same gene or to closely related genes from a gene family. We split these transcripts into genes by running a transitive clustering based on a blast search. In this clustering, transcripts, which shared at least 100 bp with a minimum identity of 97%, were considered to be isoforms of the same gene. Thus, some Trinity clusters were split into several genes. For each gene, the most expressed transcript, according to the Salmon software [64], was chosen as its representative (Fig 1).

### Technical point

The transcriptomes of one eel was used for the European eel *de novo* transcriptome assembly due to the fact that transcriptome assemblies based on multiple individuals are more prone to mistake allelic variants for recent gene duplications. Despite our transitive clustering, alleles could still be present in our transcriptome. However, these would resemble very similar paralogues, and be assigned a very low 4dTv distance. Therefore, it is implausible that local density maximum of eel paralogues found at higher 4dTv values could be alleles. Additionally, since our assessment of synteny (see *Synteny* section) is based on the genome, the paralogue pairs from which synteny can be assessed are highly unlikely to include these potential alleles.

The Atlantic salmon (*Salmo salar*) genome assembled by the International Cooperation to Sequence the Atlantic Salmon Genome [41] and the Asian arowana (*Scleropages formosus*) genome [40] were downloaded from NCBI. The genomes of zebrafish (*Danio rerio*) [65], fugu (*Takifugu rubripes*) [66], spotted gar (*Lepisosteus oculatus*) [39], and platyfish (*Xiphophorus maculatus*) [67] were downloaded from ENSEMBL (release 87). The Northern pike genome [12] was downloaded from the Northern Pike Genome web site (Genbank accession GCA_000721915.1). For each gene in the genomes, the longest transcript was chosen as the representative. For the synteny analysis, the available European [49] and Japanese [68] eel genomes were downloaded from the ZF-Genomics and the DDBJ web site, respectively (Fig 1).

### Genome and transcriptome quality assessment

In order to assess the quality of the transcriptomes and genomes, we looked for the Benchmarking set of Universal Single-Copy Orthologues (BUSCO) conserved gene set in them [69]. BUSCOs are conserved proteins which are expected to be found in complete genomes or transcriptomes. Therefore, the number of present, missing, or fragmented BUSCOs can be used as a quality control of a genome or transcriptome assembly. For this assessment, the Actinopterygii (*odb9*) gene set, which consists of 4584 single-copy genes that are present in at least 90% of Actinopterygii species, was used. As an additional comparison between the transcriptome and genomes of pike and eels, RNA-seq reads were mapped both to the genome and transcriptome assemblies using HISAT2 [70] and BWA-MEM [71] software, respectively (Fig 1), using default settings in both programs.

### Gene families

Genes were clustered into gene families by the OrthoMCL web service [72], which uses the Markov Cluster algorithm to group homologs of all the included datasets, based on all against all BLASTP searches. Therefore, the OrthoMCL gene families were also considered gene families for this study. For each gene family, a multiple protein alignment was built. To avoid transcriptome assembly artefacts, proteins longer than 1,500 amino acids, transcripts with a DUST score higher than 7 and sequences with more than 40% gaps in the alignment, were filtered out. The software Clustal Omega [73] carried out the protein multiple alignment and trimAl [74] removed the regions with too many gaps or those difficult to align. The protein alignment was used as a template to build the codon alignment by aligning the transcript sequences against the corresponding protein using the protein2dna exonerate algorithm [75] (Fig 1).

### Phylogenetic reconstruction and duplication dating

The resulting protein alignments were used by PHYLDOG [76] software to generate a species tree as well as a gene family tree corresponding to each alignment. Due to the high memory requirements of PHYLDOG, not all the gene families could be run in the same analysis, therefore 10 analyses were carried out, with 8,000 protein alignments being chosen at random for each. Once all runs were finished, we checked that the species tree topology of all the 10 species trees matched exactly. PHYLDOG uses a maximum likelihood approach to simultaneously co-estimate the species and gene family trees from all individual alignments. In order to confirm the tree topology of the PHYLDOG species tree, the species phylogeny was also reconstructed using a Bayesian approach with PhyloBayes MPI version 1.7 [77]. Furthermore, from the gene families that had one gene for each species, 100 were chosen at random to create a concatenated alignment of 43,566 amino acids. The model used was CAT-GTR and three independent MCMC chains were run for 39,872, 56,328, and 39,285 iterations (Fig 1).

PHYLDOG further tagged duplications and assigned these to specific tree branches based on the gene family trees. Between any pair of duplicated sequences, the number of transversions found in the third base of the codon was divided by the number of four-fold degenerated codons resulting in the 4dTv distance. A correction to the 4dTv was applied: ln (1–2 * distance) / -2. The 4dTv was calculated for all the duplications tagged by PHYLDOG within any gene family. These calculations are implemented by the function calculate_4dTv found in the Python scripts (S1 Material). The distribution of 4dTvs was fitted with a lognormal mixture model using the scikit-learn Gaussian Mixture class (Fig 1).

### Synteny

**Fig 2:**
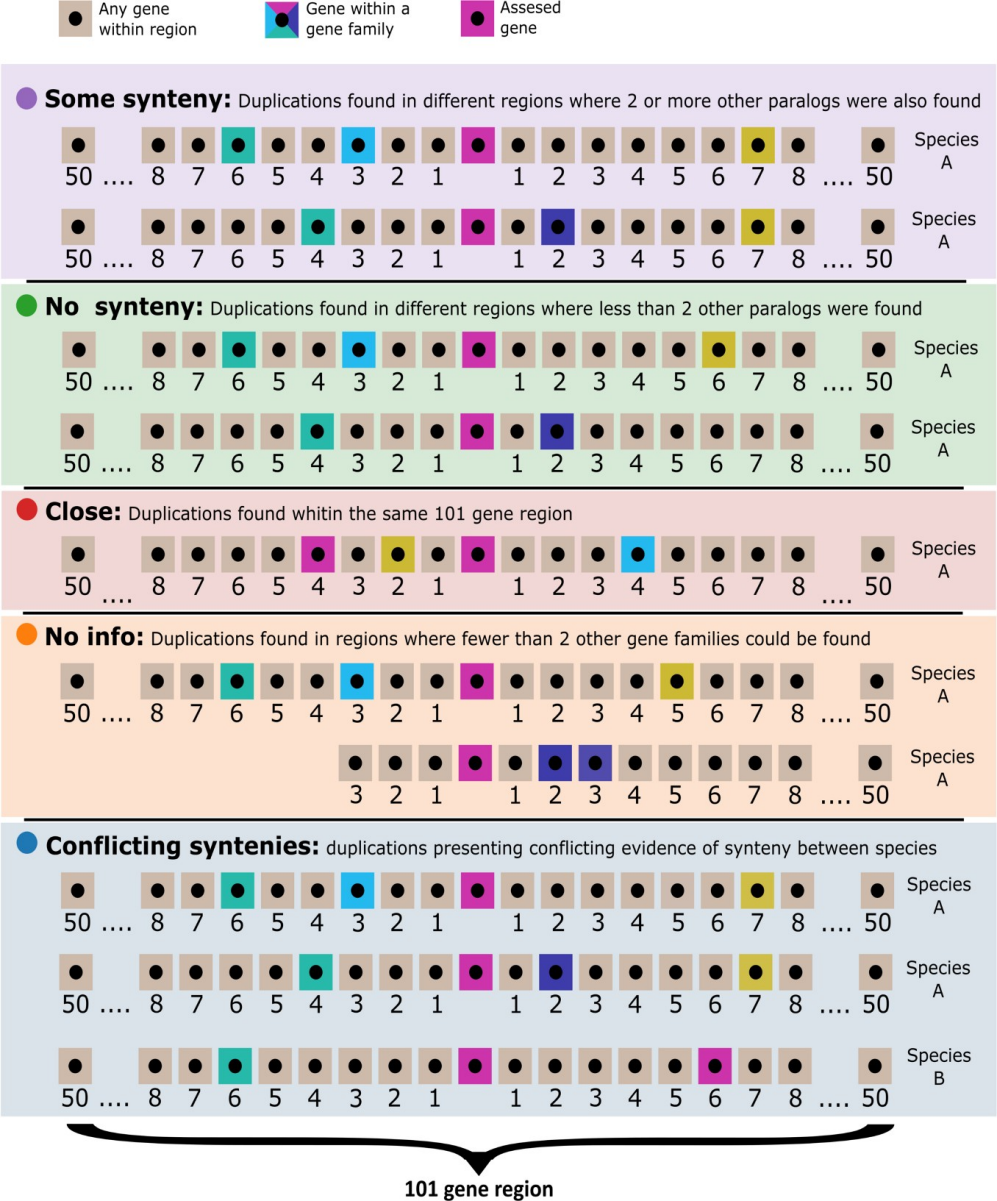
Synteny illustration. Visualization of the assigned synteny types: “some synteny” (●), paralogues of genes found close to one duplicate are also found close to the other duplicate; “no synteny” (●), less than two paralogues for other genes are found close to both paralogue duplicates; “close” (●), duplicated genes are close in the genome; “no information” (●), the duplicated genes are located in small scaffolds with too few gene families close by; “conflicting syntenies” (●), different synteny classification found in the genomes of the different species affected by the duplication. Sand coloured boxes represent genes which have not been assigned to a gene family, pink boxes represent the gene from which synteny is being assessed; all other colour boxes represent other genes which have been assigned to a gene family.

The kind of event that created each duplication was characterized by analysing the conserved synteny between the paralogues created by that duplication within a particular genome. Tandem SDs would create paralogues found close to each other in the genome, whereas the paralogues created by a WGD would be far apart, but surrounded by similar genes in each of the duplicated regions. Also, we have to consider that several phylogenetically close species can be affected by the same older duplication event. With this in mind, we categorized duplications as one of 4 classes (Fig 2): i) the paralogue genes that were found close to each other in the genome, within the 50 neighbouring genes to either side, were labelled as “close”, ii) the paralogues which were found in syntenic regions where 2 or more paralogues from other gene families were located within the 50 neighbouring genes to either side, not necessarily in the same collinear order, were labelled as “some synteny”, iii) the cases in which fewer than 2 gene families could be identified within the 50 neighbouring genes to either side, from either of the paralogues genes, were labelled as “no info”, and iv) the cases where conflicting evidence was found in the genomes of the different species affected by the duplication were labelled as “conflicting syntenies”.

This labelling of the duplications was carried out by the Python function “determine_if_pair_is_close_or_syntenic”and the Python class GenomeLocator, found in the scripts (S1 Material). The location of each gene in a genome was obtained by performing a BLAST search with its representative transcript against the genome (Fig 1).

### Investigation of functional category enrichment

The EggNOG database has gene ontology (GO) annotations for each of its gene families [78]. To match our gene families with those from the EggNOG database, the protein sequence with least gaps per each of our families was selected and a HMMER search [79] was carried out against the EggNOG position weight matrices with an e-value threshold of 10^−4^. The GO annotation of the best EggNOG hit in this search was transferred to our family. The enrichment analysis was carried out using the Fisher statistic and the weight algorithm of the topGO library [80] from the Bioconductor project. The R script go_enrichment_analysis found in the scripts (S1 Material) implements this analysis. Freshwater eel transcripts were annotated using the BlastKOALA KEGG service [81] and a Fisher exact test was carried out, using the scipy implementation, to look for overrepresented KEGG pathways in the duplications assigned to the basal freshwater eel branch (Fig 1).

## Results

### Transcriptome assemblies

Forebrain, testis, and pituitary RNA samples, from an individual European eel, were sequenced, generating a total of 191 million Illumina reads (66, 60 and 65 million from the forebrain, testis, and pituitary, respectively), with a length of 101 bp. These reads were assembled into one *de novo* transcriptome, using the Trinity assembler after a digital normalization step [61] that left 75 million representative reads. The same procedure was used to generate one *de novo* transcriptome from Illumina RNA-sequencing reads of the Japanese eel, which was downloaded from the NCBI's Sequence Read Archive [82]. The transcriptomes of Northern pike, elephantnose fish and silver arowana were also assembled by Trinity using Illumina reads from the Phylofish database [62]. The number of unigenes (henceforth referred to as transcripts) assembled ranged from 64,857 to 78,610 (Table 1) and the number of transcript clusters ranged from 46,585 to 55,667 (henceforth referred to as genes; Table 2).

**Table 1:** Included transcriptomes.

Species	N.° Reads	Q30	Transcripts	Mean GC content (%)
European eel	181,322,106	0.994	77,247	51.17
Northern Pike	553,710,218	0.989	68,489	48.05
Elephantnose fish	498,451,616	0.993	74,642	49.75
Silver arowana	490,649,254	0.992	78,610	49.18
Japanese eel	458,032,126	0.986	64,857	48.13

Metrics of included raw read datasets from European eel (*Anguilla anguilla*), Japanese eel (*Anguilla japonica*), northern Pike (*Esox lucius*), elephantnose fish (*Gnathonemus petersi*), and silver arowana (*Osteoglossum bicirrhosum*).

**Table 2:** Gene quantities far each species included.

Species	Transcripts	Genes	Representative transcripts with predicted protein	Gene family transcripts	% of genes assigned to a gene family
European eel	77,247	54,879	27,696	25,862	93.38
Japanese eel	64,857	46,585	23,780	23,098	97.13
Zebrafish	58,274	32,189	25,790	22,703	88.03
Northern pike	68,489	49,154	23,843	21,696	90.99
Elephantnose fish	74,642	50,455	24,857	22,036	88.65
Spotted gar	22,483	18,341	18,341	17,872	97.44
Silver arowana	78,610	55,667	24,938	21,604	86.63
Asian arowana	43,354	23,799	22,740	20,637	90.75
Atlantic salmon	109,584	55,104	48,593	42,625	87.72
Fugu	47,841	18,523	18,523	17,698	95.55
Platyfish	20,454	20,379	20,379	19,807	97.19

Quantities of included genes per included species: European eel (*Anguilla anguilla*), Japanese eel (*Anguilla japonica*), zebrafish (*Danio rerio*), northern pike (*Esox lucius*), elephantnose fish (*Gnathonemus petersi*), spotted gar (*Lepisosteus oculatus*), Asian arowana (*Scleropages formosus*), silver arowana (*Osteoglossum bicirrhosum*), Atlantic salmon (*Salmo salar*), fugu (*Takifugu rubripes*), and platyfish (*Xiphophorus maculatus*). “Transcripts” represents unigenes, “Genes” represents the number of transcript clusters, “Representative transcripts with predicted protein” represents the number of genes with a successful protein annotation, “Gene family transcripts” represents the representative transcripts with predicted protein with a successful gene family annotation, and “% of genes assigned to a gene family” represents the percentage of representative transcripts with predicted protein with successful gene family annotation.

### Genome and transcriptome quality

**Fig 3:**
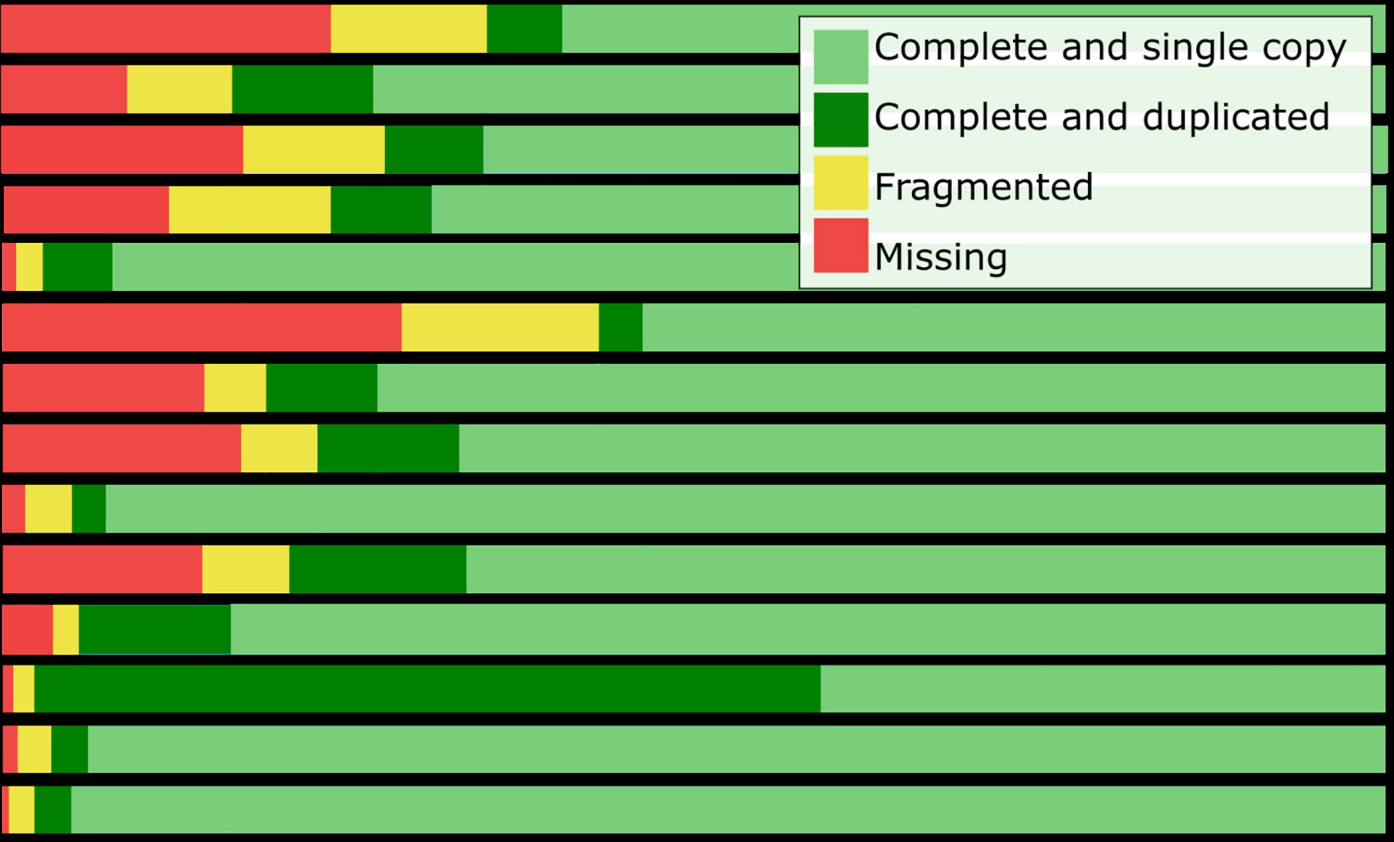
BUSCO analysis. BUSCO (Benchmarking set of Universal Single-Copy Orthologues) result for included genomes and transcriptomes. The sequence of a BUSCO gene can be found complete or fragmented in each genome and it can be found once (single copy), more than once (duplicated) or not found (missing). Included genomes are: European eel (*Anguilla anguilla*), Japanese eel (*Anguilla japonica*), Asian arowana (*Scleropages formosus*), zebrafish (*Danio rerio*), northern pike (*Esox lucius*), spotted gar (*Lepisosteus oculatus*), fugu (*Takifugu rubripes*), platyfish (*Xiphophorus maculatus*) and Atlantic salmon (*Salmo salar*). Included transcriptomes: European eel, Japanese eel, northern pike, elephantnose fish (*Gnathonemus petersii*) and silver arowana (*Osteoglossum bicirrhosum*).

The genomes and transcriptomes considered for inclusion in the analysis were quality tested by a BUSCO assessment of completeness. In general, when available, genomes were used instead of transcriptomes, except for pike, and European eel, where the transcriptomes outperformed the genomes according to the BUSCO assessment (Fig 3). Furthermore, the Japanese eel transcriptome was preferred due to a problem with the Japanese eel genome annotation. These transcriptomes also provided a higher mapping of RNA sequencing reads compared to their corresponding genomes. The percentage of reads that mapped concordantly against the genome and the transcriptome were 65.8 and 91.9%, respectively, for European eel, 74.3 and 88.4% for Japanese eel and 44.6 and 85.8% for pike. Furthermore, previously published European eel RNA-sequencing experiments were also mapped to the available European eel genome and our *de novo* transcriptome. In this case, 52.2% [83], 57.9% [84], and 66.18% [85] reads mapped concordantly against the eel genome whereas 84.3% [83], 69.5% [84], and 87.32% [85] mapped against the transcriptome.

### Gene families

Genes were assigned to gene families according to the gene family categorization of OrthoMCL [72]. The percentage of genes with predicted proteins assigned to a family by the OrthoMCL web service [72] ranged from 86.6% (silver arowana) to 97.4% (spotted gar; Table 2). Overall, 15,771 gene families were covered, from which 13,972 protein and codon alignments were built. These families contained between 2 and 172 genes, with 11 genes per family being the mode.

### Phylogenetic reconstruction and duplication characteristics

**Fig 4:**
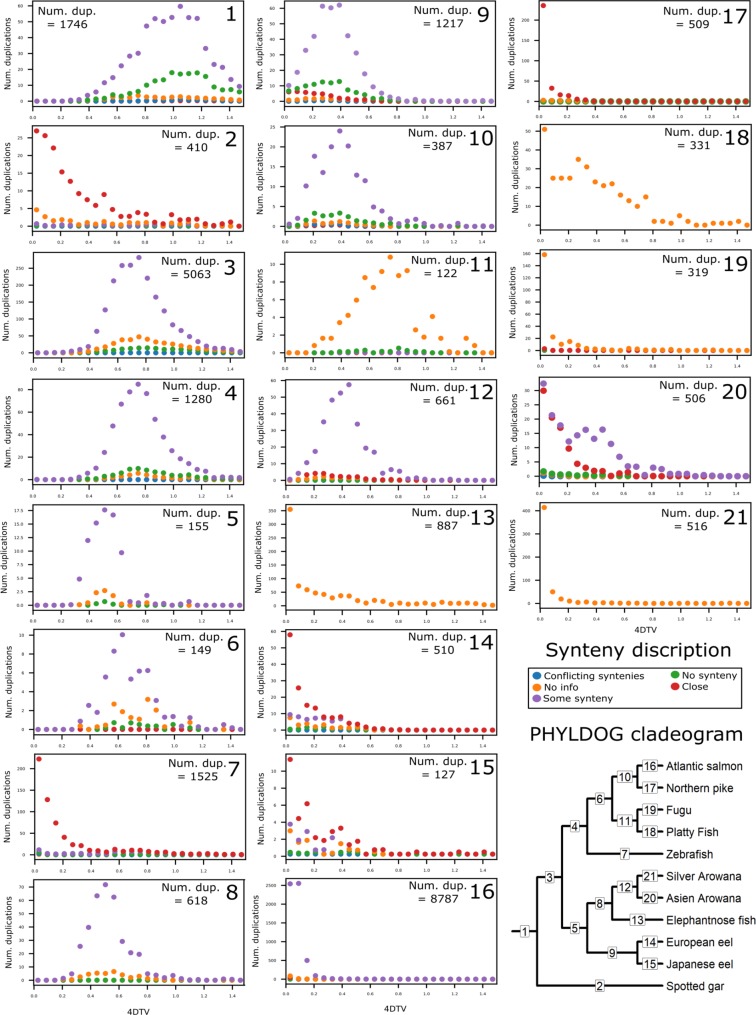
4dTv and synteny distributions of duplications per branch of the PHYLDOG species tree. Quantity, 4dTv and synteny distributions of duplications assigned to each branch of the PHYLDOG species tree. Each panel represents the branch with the corresponding number in the cladogram in the bottom right-hand corner. Species included in this study are: European eel (*Anguilla anguilla*), Japanese eel (*Anguilla japonica*), zebrafish (*Danio rerio*), northern pike (*Esox lucius*), spotted gar (*Lepisosteus oculatus*), fugu (*Takifugu rubripes*), platyfish (*Xiphophorus maculatus*), Atlantic salmon (*Salmo salar*), elephantnose fish (*Gnathonemus petersii*), Asian arowana (*Scleropages formosus*) and silver arowana (*Osteoglossum bicirrhosum*). The synteny types are the following: close (●), duplicated genes are close in the genome; some synteny (●), paralogues of genes found close to one duplicate are also found close to the other duplicate; no synteny (●), less than two paralogeus for other genes are found close to both paralogue duplicates; no information (●), the duplicated genes are located in small scaffolds with too few genes close by; conflicting syntenies (●), different synteny classifications found in the genomes of the different species affected by the duplication.

PHYLDOG software was used to tag gene duplications, create a species tree, and assign duplications to tree branches, based on gene family phylogenetic trees. Overall, trees for 10,714 gene families were created by PHYLDOG and based on the tree topology, branches in which a gene appeared to duplicate were labelled. The resulting PHYLDOG species tree matched the species tree topology created by phylobayes [77] and the resulting tree of the concatenated alignment; a cladogram of these trees is included in Fig 4. Since PHYLDOG distinguishes between gene divergence at speciation events and duplications, all genes resulting from tagged duplications are assumed to be paralogues. The assigned duplications were subsequently characterized by synteny and 4dTv distance. The 4dTv distance is used to estimate the accumulation of synonymous mutations, which can be used to estimate the time that has passed from when mutations started to accumulate. The assigned synteny classes include: “close” which indicates SDs that are a result of tandem duplications; “some synteny” which indicates a potential WGD origin (or at least a potential duplication event containing >100 genes); and “no synteny”, which supports neither a SD nor a WGD origin (Fig 2).

PHYLDOG labelled 5,063 duplications to the basal teleost branch, after the split of the spotted gar, with a 4dTv mode of 0.75 (Fig 4, Node 3). Of the paralogues created by these duplications, 73.8% were located in regions with some synteny, 1.5% were close to each other, and 22.5% had no synteny (Fig 4, Node 3). These percentages were calculated without taking into account the duplications where no information regarding the physical location of the genes could be established. The duplications assigned to this basal teleost branch (Fig 4, Node 3) included all gene families with members in both sister clades and thus are assumed to have originated at the 3R. This branch further included hundreds of duplications found in the eels. From these duplications, 62 families had conserved 2 paralogue pairs, one of which had started to diverge at 3R and one in a common ancestor of freshwater eels after the split with osteoglossomorphs. From the paralogue pairs, which had started to diverge in a common ancestor of freshwater eels (Fig 4, Node 9), from these 62 families; 30 were located in regions with some synteny, 9 were close to each other, 11 had no synteny and for 12 pairs no information regarding the physical location of the genes could be established.

1,280 duplications were assigned to the branch basal to the included Clupeocephalan teleosts: zebrafish, fugu, platyfish, northern pike, and Atlantic salmon (Fig 4, Node 4). These duplications showed a very similar distribution with those of the 3R branch, with an overall 4dTv mode of 0.75 (Fig 4, Node 3).

The basal freshwater eel branch was assigned 1,217 duplications of which 55.3, 15.8, and 24.3% were labelled as some synteny, close and without synteny, respectively (Fig 4, Node 9). The European and Japanese eel specific branches were assigned 510 and 127 duplications, from which 32.2, and 34.7% were labelled as some synteny, 50.0 and 48.4% were labelled as close, and 17.1 and 14.7% were labelled as without synteny, respectively (Fig 4, Nodes 14 and 15).

The basal Osteoglossomorpha and the basal arowana branches were assigned 618 and 661 duplications, from which 95.7, and 76.2% were labelled as some synteny, 0.9 and 17.7% were labelled as close, and 3.5 and 5.1% were labelled as without synteny, respectively (Fig 4, Nodes 8 and 12).

The salmon and zebrafish specific branches were assigned 8,787 and 1,525 duplications, respectively, and most of these duplications seemed recent, according to their 4dTv distances. In the salmon branch, most of the duplications (87.0%) were characterized by paralogues located in syntenic regions, whereas most of the zebrafish paralogues (60.5%) were characterized as “close” (Fig 4, Nodes 16 and 7).

For all the included species the “close” paralogues (tandem SDs) tended to show low divergence according to their 4dTv, whereas the duplications found in synteny and most of the duplications without sufficient genomic location information, were more often found to have higher 4dTv distance (Fig 4).

**Fig 5:**
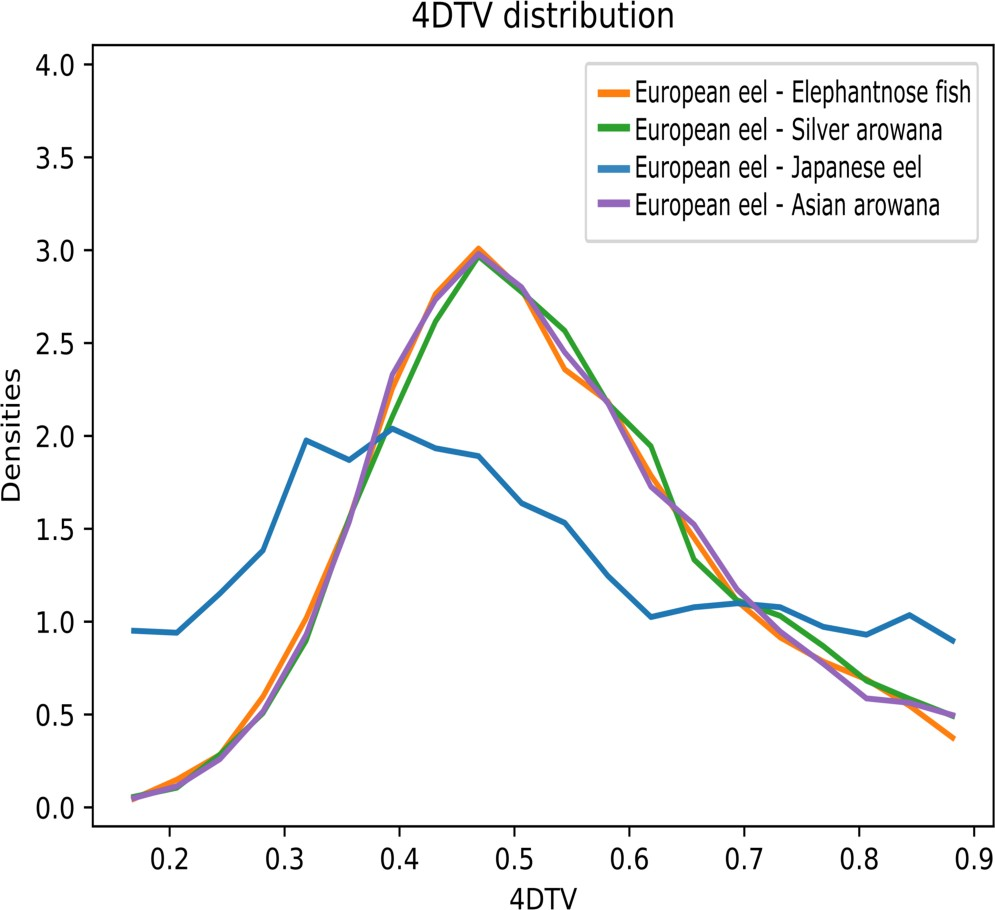
4dTv distribution between European eel, elephantnose fish, and the arowanas homologs. 4dTv distribution of European eel (*Anguilla anguilla*) and Japanese eel homologs (▬), European eel and elephantnose fish (*Gnathonemus petersii*) homologs (▬), and European eel, silver arowana (*Osteoglossum bicirrhosum*) homologs (▬), and European eel and Asian arowana (*Scleropages formosus*) homologs (▬).

**Fig 6:**
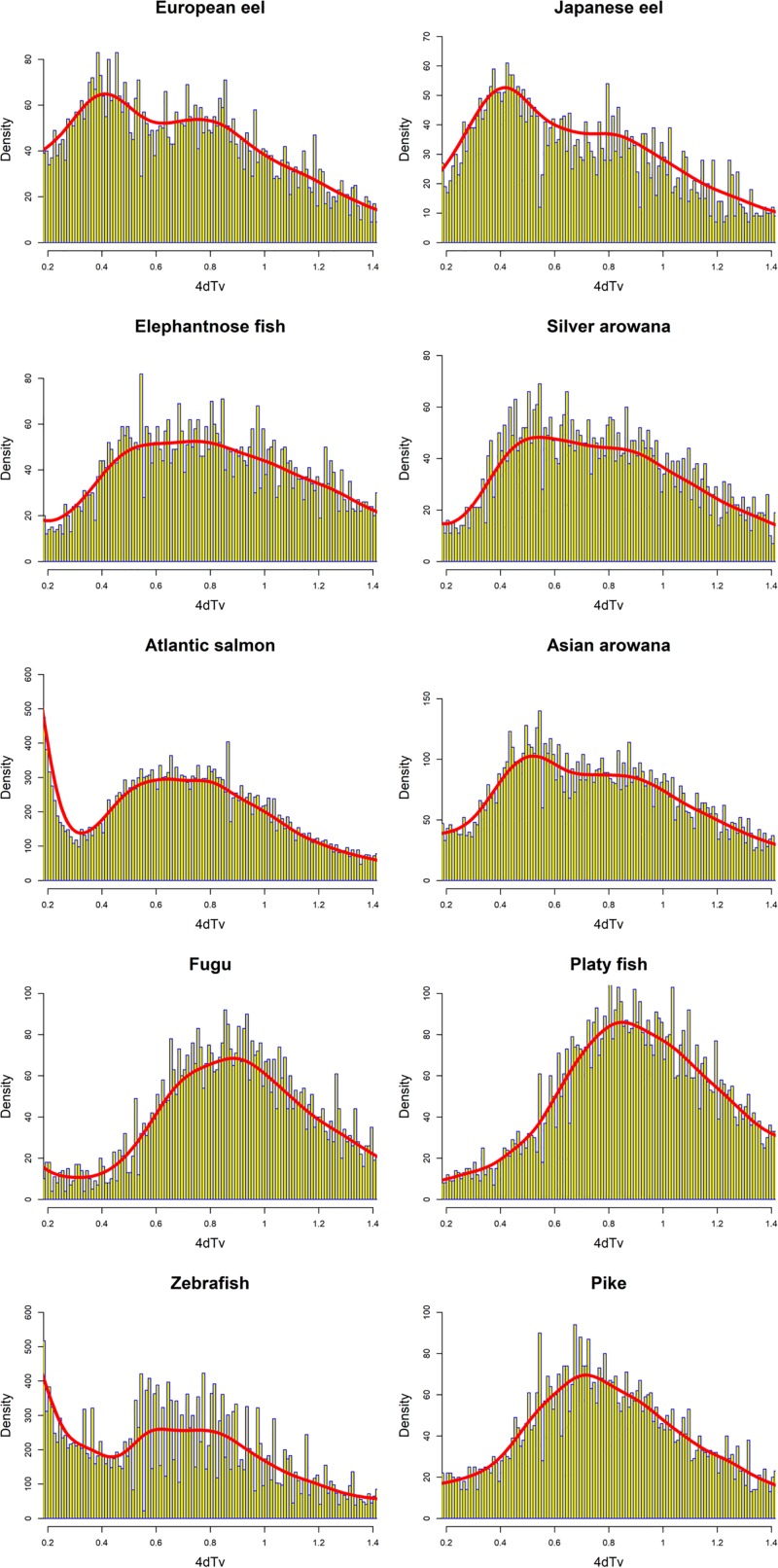
Density distribution of all 4dTv distances between teleost paralogues. Histograms of all 4dTv distances between paralogues of the included teleosts, presented with yellow and blue bars. Furthermore, a probability density estimate curve is plotted on top of the histograms in red. Density values (y-axis) do not correspond to the density estimate. The included species are: European eel (*Anguilla anguilla*), Japanese eel (*Anguilla japonica*), zebrafish (*Danio rerio*), northern pike (*Esox lucius*), spotted gar (*Lepisosteus oculatus*), fugu (*Takifugu rubripes*), platyfish (*Xiphophorus maculatus*), Atlantic salmon (*Salmo salar*), elephantnose fish (*Gnathonemus petersii*), Asian arowana (*Scleropages formosus*) and silver arowana (*Osteoglossum bicirrhosum*).

The duplications assigned to the basal freshwater eel branch showed a 4dTv mode of ~0.4 (Fig 4, Node 9). In order to investigate the relative age of all the homolog pairs found in the eels, we ran a 4dTv distance analysis independent of the PHYLDOG tree topology. In this analysis, we compared the 4dTv distribution found for European eel homologs with Japanese eel, elephantnose fish, silver arowana and Asian arowana (Fig 5). The results showed a homolog density mode at 4dTv of ~0.4 for the European and Japanese eel, and 0.5 for the speciation event that separated elephantnose fish, silver arowana, and Asian arowana from the freshwater eels (Fig 5). Furthermore, in order to obtain comparisons between older eel paralogues (>0.2 of 4dTv) and other teleosts, we produced histograms of the 4dTv distances calculated between all paralogues within each species from 0.2 to 1.4 of 4dTv (Fig 6). Additionally, a nonparametric probability density estimate was calculated, using the Gaussian mixture model and plotted on top of the histogram (Fig 6). These results show an older local density maximum (likely originating from 3R) for all teleosts ranging from 0.62 (Zebrafish) to 0.88 (Fugu) of 4dTv. Furthermore, European eel, Japanese eel, Asian arowana and possibly silver arowana showed an additional 4dTv local density maximum at 0.41, 0.42, 0.56 and 0.55 of 4dTv, respectively (Fig 6). A more recent local density maximum was seen in the Atlantic salmon distribution at 0.15 (Fig 4, Node 16).

### Functional category enrichment

To investigate whether some functional categories were overrepresented among the paralogues assigned to the basal eel branch, two enrichment tests were carried out. First, 1,041 unique GO terms were assigned to 3,607 genes from the basal eel branch, by comparing them to the annotated EggNOG gene families. The full GO annotation can be found in S1 Table. From these terms, we performed an enrichment analysis using the topGO R library [80]. The resulting enriched GO-terms are presented in Table 3. In many cases, these terms were involved either in signalling (e.g. receptor activity, molecular transducer activity, or small GTPase mediated signal transduction), development (e.g. embryonic camera-type eye morphogenesis, gastrulation with mouth forming second, or cell migration involved in gastrulation), ion transport (e.g. anion binding, ATP hydrolysis coupled proton transport, or organic anion transmembrane transporter activity), metabolism (e.g. carbohydrate phosphorylation, ubiquitin-dependent protein catabolic process, or lipopolysaccharide biosynthetic process), or neuronal function (e.g. forebrain development, motor neuron axon guidance, or neuromast development; Table 3). Secondly, KEGG terms were assigned to 1,674 freshwater eel genes using BlastKOALA [81] and mapped onto the KEGG pathways using the KEGG Mapper tool. A Fisher test, corrected for multiple comparisons using False Discovery Rate, was used to look for enriched KEGG pathways in the basal eel branch (Table 4). Most of the KEGG pathways found to be enriched were related to the immune system, nervous system, metabolism and signal transduction. Interestingly, the most significantly enriched KEGG pathway was “Dopaminergic synapse”.

**Table 3:** Enriched Go-terms from the shared freshwater eel branch of Fig 4.

Aspect	GO ID	Term	Annotated	Significant	Expected	FDR
Biological Process	GO:0007264	small GTPase mediated signal transductio …	256	43	22.97	0.000064
Biological Process	GO:0045176	apical protein localization	3	3	0.27	0.00072
Biological Process	GO:0008045	motor neuron axon guidance	10	5	0.9	0.00099
Biological Process	GO:0048514	blood vessel morphogenesis	121	20	10.86	0.00257
Biological Process	GO:0000132	establishment of mitotic spindle orienta …	8	4	0.72	0.00335
Biological Process	GO:0048596	embryonic camera-type eye morphogenesis	11	4	0.99	0.00625
Biological Process	GO:0015991	ATP hydrolysis coupled proton transport	20	6	1.79	0.00661
Biological Process	GO:0008333	endosome to lysosome transport	2	2	0.18	0.00804
Biological Process	GO:0015031	protein transport	284	39	25.48	0.00900
Biological Process	GO:0006886	intracellular protein transport	156	19	14	0.00907
Biological Process	GO:0007160	cell-matrix adhesion	16	5	1.44	0.01084
Biological Process	GO:0001756	somitogenesis	50	10	4.49	0.01200
Biological Process	GO:0060042	retina morphogenesis in camera-type eye	37	9	3.32	0.01887
Biological Process	GO:0072358	cardiovascular system development	280	44	25.12	0.01905
Biological Process	GO:0040023	establishment of nucleus localization	4	3	0.36	0.02262
Biological Process	GO:0009826	unidimensional cell growth	3	2	0.27	0.02268
Biological Process	GO:0030326	embryonic limb morphogenesis	3	2	0.27	0.02268
Biological Process	GO:0008202	steroid metabolic process	34	3	3.05	0.02280
Biological Process	GO:0007179	transforming growth factor beta receptor …	13	4	1.17	0.02382
Biological Process	GO:0071840	cellular component organization or bioge …	846	81	75.91	0.02822
Biological Process	GO:0048884	neuromast development	15	4	1.35	0.02854
Biological Process	GO:0001569	patterning of blood vessels	8	3	0.72	0.02858
Biological Process	GO:0016998	cell wall macromolecule catabolic proces …	8	3	0.72	0.02858
Biological Process	GO:0046835	carbohydrate phosphorylation	8	3	0.72	0.02858
Biological Process	GO:0043473	pigmentation	57	7	5.11	0.02863
Biological Process	GO:0060059	embryonic retina morphogenesis in camera …	14	4	1.26	0.03104
Biological Process	GO:0001702	gastrulation with mouth forming second	23	6	2.06	0.04241
Biological Process	GO:0060034	notochord cell differentiation	6	3	0.54	0.04259
Biological Process	GO:0061035	regulation of cartilage development	7	3	0.63	0.04260
Biological Process	GO:0009103	lipopolysaCellular Componentharide biosynthetic process	4	2	0.36	0.04268
Biological Process	GO:0043114	regulation of vascular permeability	4	2	0.36	0.04268
Biological Process	GO:0015721	bile acid and bile salt transport	4	2	0.36	0.04268
Biological Process	GO:0006511	ubiquitin-dependent protein catabolic pr …	91	14	8.17	0.04728
Biological Process	GO:0030900	forebrain development	53	10	4.76	0.04832
Biological Process	GO:0042074	cell migration involved in gastrulation	37	9	3.32	0.04885
Cellular Component	GO:0031105	septin complex	6	4	0.54	0.00084
Cellular Component	GO:0030018	Z disc	10	5	0.9	0.00099
Cellular Component	GO:0031461	cullin-RING ubiquitin ligase complex	28	8	2.52	0.00555
Cellular Component	GO:0008290	F-actin capping protein complex	2	2	0.18	0.00807
Cellular Component	GO:0005915	zonula adherens	2	2	0.18	0.00807
Cellular Component	GO:0005737	cytoplasm	1750	175	157.31	0.01734
Cellular Component	GO:0005768	endosome	47	8	4.22	0.01771
Cellular Component	GO:0033180	proton-transporting V-type ATPase V1 do …	10	4	0.9	0.01913
Cellular Component	GO:0030424	axon	7	3	0.63	0.01920
Cellular Component	GO:0000159	protein phosphatase type 2A complex	3	2	0.27	0.02275
Cellular Component	GO:0005667	transcription factor complex	79	12	7.1	0.02336
Cellular Component	GO:0005912	adherens junction	7	4	0.63	0.04255
Cellular Component	GO:0031519	PcG protein complex	9	4	0.81	0.04258
Cellular Component	GO:0005890	sodium:potassium-exchanging ATPase compl …	4	2	0.36	0.04281
Cellular Component	GO:0043198	dendritic shaft	4	2	0.36	0.04281
Cellular Component	GO:0005885	Arp2/3 protein complex	4	2	0.36	0.04281
Cellular Component	GO:0005765	lysosomal membrane	16	4	1.44	0.04914
Molecular Function	GO:0005525	GTP binding	251	43	22.24	1.6e-05
Molecular Function	GO:0043168	anion binding	1289	142	114.24	0.0018
Molecular Function	GO:0060089	molecular transducer activity	778	56	68.95	0.0078
Molecular Function	GO:0004331	fructose-2 6-bisphosphate 2-phosphatase …	2	2	0.18	0.0078
Molecular Function	GO:0045296	cadherin binding	2	2	0.18	0.0078
Molecular Function	GO:0046933	proton-transporting ATP synthase activit …	10	4	0.89	0.0083
Molecular Function	GO:0004702	receptor signaling protein serine/threon …	19	7	1.68	0.0112
Molecular Function	GO:0008242	omega peptidase activity	6	3	0.53	0.0113
Molecular Function	GO:0031683	G-protein beta/gamma-subunit complex bin …	6	3	0.53	0.0113
Molecular Function	GO:0008013	beta-catenin binding	7	3	0.62	0.0185
Molecular Function	GO:0016820	hydrolase activity acting on acid anhyd …	38	8	3.37	0.0219
Molecular Function	GO:0004749	ribose phosphate diphosphokinase activit …	3	2	0.27	0.0221
Molecular Function	GO:0008601	protein phosphatase type 2A regulator ac …	3	2	0.27	0.0221
Molecular Function	GO:0003796	lysozyme activity	3	2	0.27	0.0221
Molecular Function	GO:0008146	sulfotransferase activity	43	10	3.81	0.0249
Molecular Function	GO:0051287	NAD binding	22	5	1.95	0.0298
Molecular Function	GO:0003714	transcription corepressor activity	9	3	0.8	0.0388
Molecular Function	GO:0008514	organic anion transmembrane transporter …	22	5	1.95	0.0399
Molecular Function	GO:0004872	receptor activity	695	41	61.59	0.0495

Enriched Go-terms from the duplicated genes shared by freshwater eels. “Aspects” indicates the specific GO-term aspect of each enriched GO-term. “GO ID” indicates the identification number of each enriched GO-term. “Term “indicates the verbal description of each enriched GO-term. “Annotated” indicates the number of GO-terms which are associated with each enriched GO-term. “Significant” indicates the number of GO-terms associated to each enriched GO-term found among the duplicated genes. “Expected” indicates the number of GO-terms expected to be found linked to each enriched GO-term. “FDR” indicates the False Discovery Rate adjusted P-value from the Fisher exact test of enrichment.

**Table 4:** Enriched KEGG-terms from the shared freshwater eel branch of Fig 4.

KEGG ID	Term	Annotated	Significant	Expected	FDR
04728	Dopaminergic synapse	38	283	12	0,000001
03015	mRNA surveillance pathway	21	129	5	0,000082
04660	T cell receptor signaling pathway	27	204	8	0,000082
04071	Sphingolipid signaling pathway	29	238	10	0,000112
05142	Chagas disease (American trypanosomiasis)	23	180	7	0,000518
04659	Th17 cell differentiation	23	184	7	0,000596
05162	Measles	21	168	7	0,001190
04390	Hippo signaling pathway	29	282	11	0,001190
04658	Th1 and Th2 cell differentiation	19	148	6	0,001601
04261	Adrenergic signaling in cardiomyocytes	28	291	12	0,004378
05100	Bacterial invasion of epithelial cells	20	183	7	0,005845
05032	Morphine addiction	18	155	6	0,005845
00625	Chloroalkane and chloroalkene degradation	5	10	0	0,005845
04640	Hematopoietic cell lineage	12	79	3	0,006536
04910	Insulin signaling pathway	26	276	11	0,006551
04630	Jak-STAT signaling pathway	18	171	7	0,012920
04016	MAPK signaling pathway—plant	6	22	1	0,014553
04022	cGMP-PKG signaling pathway	29	350	14	0,015915
04917	Prolactin signaling pathway	15	133	5	0,015915
05130	Pathogenic Escherichia coli infection	13	108	4	0,019059
05418	Fluid shear stress and atherosclerosis	22	245	10	0,022738
00020	Citrate cycle (TCA cycle)	8	47	2	0,022808
04080	Neuroactive ligand-receptor interaction	31	395	16	0,023393
04151	PI3K-Akt signaling pathway	37	499	20	0,023393
04514	Cell adhesion molecules (CAMs)	21	231	9	0,023393
04391	Hippo signaling pathway—fly	16	158	6	0,034750
05340	Primary immunodeficiency	7	41	2	0,036069
04350	TGF-beta signaling pathway	16	164	7	0,038388
05133	Pertussis	12	111	5	0,045104
05152	Tuberculosis	21	247	10	0,047421
04664	Fc epsilon RI signaling pathway	12	113	5	0,048089
00510	N-Glycan biosynthesis	9	71	3	0,048943
04144	Endocytosis	37	533	22	0,048943
00350	Tyrosine metabolism	6	34	1	0,048943
04510	Focal adhesion	28	379	15	0,048943

Enriched KEGG-terms from the duplicated genes shared by freshwater eels. “KEGG ID” indicates the identification number of each enriched KEGG pathway. “Term”indicates the verbal description of each enriched KEGG pathway. “Annotated” indicates the number of KEGG pathways, which are associated with each enriched KEGG pathway. “Significant” indicates the number of KEGG pathways associated with each enriched KEGG pathway found among the duplicated genes. “Expected” indicates the number of KEGG pathways expected to be found associated with each enriched KEGG pathway. “FDR” indicates the False Discovery Rate adjusted P-value from the Fisher exact test of enrichment.

## Discussion

The present study found more than one thousand gene families in which the gene family tree topology indicates a duplication in a common ancestor of freshwater eels sometime after the split of Elopomorpha and Osteoglossomorpha. Only phylogenetic species tree branches with previously documented WGDs (Fig 4, Nodes 1, 3, 4, and 16) and the zebrafish specific branch (Fig 4, Node 7) were assigned more duplications than the basal freshwater eel branch (Fig 4, Node 9). The vast majority of the assigned zebrafish specific duplications formed a 4dTv local density maximum at ~0 and were found “close” in the genome, thus these duplications appear to be tandem SDs, the presence of which concurs with previous studies [8,11,65].

### The origin of the duplications assigned to the basal freshwater eel branch

In some cases, it has been shown that SDs could be retained at specific points in time [5,15,16]. However, most duplications assigned to the basal freshwater eel branch were detected in large syntenic blocks which opposes a hypothesis of a SD origin.

Rather the synteny results suggest that the duplications assigned to the basal freshwater eel branch originated in larger portions e.g. whole regions (large SDs), chromosomes or genomes. In particular, a WGD origin is consistent with the number of duplications observed and the 4dTv distribution (Fig 4, Node 9), which showed one distinct 4dTv density mode (4dTv ~0.4) placed along the long branch leading to the freshwater eels. Fig 6 (and Fig 5) further shows duplications which started diverging at the 3R in the eel transcriptome, as a 4dTv local density maximum of ~0.75. The notable similarity between this local density maximum and the local density maximum of the other 3R generated genes from all the included teleosts (Figs 4 and 6) suggests that these paralogues (4dTv ~ 0.75) were created by the 3R. This hypothesis is further supported by the results of the phylogenetic analysis (Fig 4, Nodes 3), which assigned hundreds of duplications, which are still present in the eel, to the 3R branch. Moreover, no modes of comparable magnitude at 4dTv ~ 0.75 can be seen in any of the post 3R branches leading to the freshwater eels (Fig 4, Nodes 5 and 9). Therefore, if the duplications assigned to the basal freshwater eel branch were created by a WGD event, and assuming instant rediploidization after the 3R, this event would be more recent and different to the 3R, and thus should be named a 4R WGD event.

However, cytological rediploidization is not always completed immediately after an autotetraploidization WGD event, as shown in the case of salmonids [43]. Therefore, the origin of the duplications assigned to the basal freshwater eel branch could also be explained by a hypothesis of lineage-specific rediploidization after the 3R. Protracted rediploidization could result in lower rates of gene losses since deleterious mutations have less time to accumulate in one paralogue and thereby create a pseudogene, which could explain the high number of paralogue pairs found in eel. This hypothesis could also explain both the PHYLDOG and 4dTv results, as paralogue genes only start to diverge after the rediploidization of their genomic region [41,43,44]. If the duplications assigned to the basal freshwater eel branch had, in fact, experienced delayed rediploidization from the 3R, the same genomic regions would have also experienced delayed rediploidization in the lineage of the remaining teleosts. Interestingly, relatively large quantities of duplications, with conserved synteny, were also assigned to the basal Clupeocephala branch and the Osteoglossomorpha branches (Fig 4, Nodes 4, 8, and 12). This observation supports the hypothesis that the duplications assigned to the basal freshwater eel branch were located in genomic regions, which experienced delayed rediploidization after the 3R. However, lineage-specific rediploidization has only been unequivocally documented in salmonids, and more studies are needed to demonstrate this process in other species. Therefore, it remains to be determined if this mechanism is a salmonid specific phenomenon. Furthermore, due to the observed 4dTv distances, the mechanisms would have protracted rediploidization for a longer time in eels than in salmonids. Moreover, the 4dTv analysis revealed very similar results for the 3R branch and the basal Clupeocephala branch (Fig 4, Nodes 3 and 4) and for the shared Osteoglossomorpha and Arowana branches, respectively (Fig 4, Nodes 8 and 12). This result supports the hypothesis that these duplications were divided due to a potential PHYLDOG artefact, explained below. Additionally, 62 gene families were found which showed a topology concurring with a 3R event followed by a 4R event, since duplication had been conserved from both events in the same gene family. From these 62 families, 30 paralogues pairs of the suspected 4R duplications were located in regions with some synteny. These trees directly oppose the hypothesis of protracted rediploidization; however, only for these 30 families. In the event of an eel 4R WGD, more such trees would be expected.

### Possible PHYLDOG artefact

According to PHYLDOG, the shared teleost duplications split into two events placed in the 3R branch and the basal Clupeocephala branch (Fig 4, Nodes 3 and 4). These results could be caused by an artefact from the phylogenetic analysis. Specifically, PHYLDOG software assigns duplications to branches based on the successful identification of the daughter genes on the branches of both sister clades, thus lower genomic information (fewer or less complete genomes/transcriptomes) increases the chance of not finding a gene and thus misplacing duplications. In this case, although the number of genomes/transcriptomes are the same, the amount of genomic information is substantially different between the two daughter clades, since the genomes/transcriptomes on one side (Fig 4, from Node 5), are generally much less complete than those on the other side (Figs 3 and 4, from Node 4). In support of this hypothesis is the 4dTv analysis, in which the duplications assigned to the 3R and the basal Clupeocephala branch indicate approximately the same mode (Fig 4, Nodes 3 and 4), suggesting that they started to diverge at the same time. Therefore, a PHYLDOG artefact, in which duplications can leak down to a daughter branch which is basal to a clade containing more genomic information, is also a parsimonious explanation for most of the duplications assigned to the basal Clupeocephala branch.

### Arowana results

As an unexpected result of our analysis, the included Osteoglossomorphas also appear to contain a high quantity of duplications, which likely started diverging after the split between Elopomorphas and Osteoglossomorphas. These duplications also included a high occurrence of paralogues with some conserved synteny between them. This result suggests that these genes were duplicated in larger portions e.g. whole regions (large SDs), chromosomes or genome and not by smaller SDs. When combining the basal Osteoglossomorpha and the basal arowana branches these were assigned a similar quantity of duplications as the basal freshwater eel branch. This result supports the hypothesis that some genomic regions were still under tetrasomic inheritance, from the 3R, at the time of the split between Elopomorphas and Osteoglossomorphas. However, it is also possible that the duplications were generated by a separate duplication event in a common ancestor to the included Osteoglossomorphas but have leaked into the basal arowana branch and the Asian arowana specific branch in the phylogenetic analysis due to the PHYLDOG artefact described above. The PHYLDOG artefact hypothesis is supported by the 4dTv analysis, as the 4dTv modes of these branches are very similar, and since the Elephantnose fish transcriptomes are the least complete dataset of these branches. To generate a better supported hypothesis of the origin of these duplications a study dedicated to this purpose should be conducted.

### Start of divergence of the duplications assigned to the basal freshwater eel branch

In the independent 4dTv analysis, without considering phylogenetic tree topologies, the 4dTv of the homologs between the European eel and the Japanese eel, the elephantnose fish and the arowanas, showed that European eel and Japanese eel homologs have a 4dTv mode at ~ 0.4. On the other hand, the homologs between the European eel and any Osteoglossomorpha species form a 4dTv mode at ~ 0.5 (Fig 5). This result indicates that the duplications found in the freshwater eel species started diverging after the split between Elopomorphas and Osteoglossomorphas (Fig 5). Therefore, the phylogenetic reconstruction and the 4dTv distances together suggest that the duplications assigned to the basal freshwater eel branch (4dTv ~ 0.4) started diverging after the teleost specific 3R duplication event (320–350 MYA) [35,36] and after the split between eels and Osteoglossomorphas, but before the Ss4R (88–103 MYA) [86].

If the 4dTv mode observed in the basal freshwater eel branch was the result of new duplications, then these duplications would likely have originated in a common ancestor to all members of the anguillidae family, as these first appear 20–50 MYA [87]. Due to the 4dTv observed, this event could also be shared by wider Elopomorpha; however, without analysing other anguilliforms or Elopomorpha transcriptomes or genomes, this hypothesis remains speculative.

### Previously published related data

In concurrence with the present study, other studies have reported data suggesting an unusually high quantity of gene duplications in eels. In the additional data included by Inoue *et al*. [27], the eel and zebrafish are the species with the highest percentage of duplicated genes (36.6% and 31.9%, respectively). Furthermore, an unexpectedly high number of Hox genes (73 genes) were found in the analysis of the draft eel genome [49]. In this study [49], the phylogenetic distance between Hox clusters was remarkably short, making it impossible to distinguish between the 3R “a” or “b” association of 3 out of 4 cluster pairs based on DNA sequence alone. Several other studies focusing on particular genes have likewise found paralogue pairs in eels, which are not found in other teleosts [48–56] and similarly, an unexpected short phylogenetic distance is often found between eel paralogue pairs. These results support both a 4R hypothesis and a hypothesis of a 3R origin followed by protracted rediploidization. However, many of the referenced studies also presented results of weak conserved local synteny indicating a 3R origin. These synteny results are unexpected following both a 4R hypothesis and the hypothesis of protracted rediploidization. We draw this conclusion based on the notion that the close genomic region of genes, which experienced delayed rediploidization, is highly expected to also have been under tetrasomic inheritance for an extended period [43]. Thus, these neighbouring genes should not accumulate mutations similarly to homolog regions of other teleosts, which experienced immediate rediploidization, and thus the synteny of these regions are unlikely to match.

## Conclusions

The data presented in this study support the hypothesis that a remarkably high amount of paralogues pairs started to diverge in a common ancestor of the freshwater eel lineage after the split from the Osteoglossomorpha lineage. The 4dTv and phylogenetic analyses revealed a clear clustering of these paralogues in the basal freshwater eel branch with a 4dTv mode at ~0.4. The synteny of these paralogue pairs suggests they originated in large portions, most likely from a WGD event. However, the results do not unequivocally support/oppose whether i) These paralogues originated from the 3R but are located in genomic regions which have experienced protracted rediploidization; ii) These paralogues originated in a 4R WGD in a common ancestor to freshwater eels; or iii) Both i and ii have contributed to the evolution of these paralogues. The present results offer robust information on the duplicated gene complement of freshwater eels, thus providing novel insights into the peculiar biology of the critically endangered European eel. However, additional high quality genome resources of other Elopomorpha members are needed to further study the dynamics of gene duplication and conservation in early teleost evolution.

## Supporting information

S1 Table GO annotation of eel duplication gene families from EggNOG. GO annotation from EggNOG of eel duplication gene families shared by freshwater eels. Including: “Orthogroup”, “Descriptions”, “Functional annotations”, “GO categories”, and “GO IDs”.
(CSV)

S1 Material All scripts used for analysis. (ZIP)
